# Symptomatic and restorative therapies in neuromyelitis optica spectrum disorders

**DOI:** 10.1007/s00415-021-10783-4

**Published:** 2021-09-05

**Authors:** Hesham Abboud, Andrea Salazar-Camelo, Naveen George, Sarah M. Planchon, Marcelo Matiello, Maureen A. Mealy, Andrew Goodman, On-behalf of the Guthy-Jackson Foundation NMO International Clinical Consortium, Raed Alroughani, Ayse Altintas, Metha Apiwattanakul, Nasrin Asgari, Renata Barbosa Paolilo, Jeffrey Bennett, Denis Bernardi Bichuetti, Terrence F. Blaschke, Alexey Boyko, Simon Broadley, Edgar Carnero Contentti, Jeffrey A. Cohen, Guillermo Delgado-Garcia, Irena Dujmovic Basuroski, Jose Flores-Rivera, Kazuo Fujihara, Joachim Havla, Kerstin Hellwig, Jyh Yung Hor, Saif Huda, Raffaele Iorio, Sven Jarius, Dorlan Kimbrough, Ilya Kister, Ingo Kleiter, Najib Kissani, Marco Lana-Peixoto, Maria Isabel Leite, Michael Levy, Youssoufa Maiga, Yang Mao-Draayer, Sara Mariotto, Esther Melamed, Veronika E. Neubrand, Celia Oreja-Guevara, Jacqueline Palace, Anne-Katrin Pröbstel, Peiqing Qian, Chao Quan, Claire Riley, Marius Ringelstein, Maria Jose Sa, Sasitorn Siritho, Terry J. Smith, Ibis Soto de Castillo, Silvia Tenembaum, Pablo Villoslada, Jens Wuerfel, Dean Wingerchuk, Bassem Yamout, Michael Yeaman

**Affiliations:** 1 Multiple Sclerosis and Neuroimmunology Program, Parkinson’s and Movement Disorders Center, University Hospitals of Cleveland, Case Western Reserve University, Bolwell, 5th floor, 11100 Euclid Avenue, Cleveland, OH 44106 USA; 2 Department of Neurology, Johns Hopkins University School of Medicine, Baltimore, MD USA; 3 The Mellen Center for Multiple Sclerosis, Cleveland Clinic, Cleveland, OH USA; 4 Neurology Department, Massachusetts General Hospital, Harvard Medical School, Boston, MA USA; 5 Horizon Therapeutics Plc, Deerfield, IL USA; 6 Neuroimmunology Division, Department of Neurology, University of Rochester Medical Center, Rochester, NY USA

**Keywords:** NMOSD, Neuromyelitis optica, NMO, Symptomatic, Neuropathic pain, Tonic spasms, Remyelination, Mesenchymal stem cells

## Abstract

Neuromyelitis optica spectrum disorders (NMOSD) are a group of autoimmune inflammatory conditions that primarily target the optic nerves, spinal cord, brainstem, and occasionally the cerebrum. NMOSD is characterized by recurrent attacks of visual, motor, and/or sensory dysfunction that often result in severe neurological deficits. In recent years, there has been a significant progress in relapse treatment and prevention but the residual disability per attack remains high. Although symptomatic and restorative research has been limited in NMOSD, some therapeutic approaches can be inferred from published case series and evidence from multiple sclerosis literature. In this review, we will discuss established and emerging therapeutic options for symptomatic treatment and restoration of function in NMOSD. We highlight NMOSD-specific considerations and identify potential areas for future research. The review covers pharmacologic, non-pharmacologic, and neuromodulatory approaches to neuropathic pain, tonic spasms, muscle tone abnormalities, sphincter dysfunction, motor and visual impairment, fatigue, sleep disorders, and neuropsychological symptoms. In addition, we briefly discuss remyelinating agents and mesenchymal stem cell transplantation in NMOSD.

**Supplementary Information:**

The online version contains supplementary material available at 10.1007/s00415-021-10783-4.

## Introduction

Immune-modulating therapies for relapse prevention of neuromyelitis optica spectrum disorders (NMOSD) have evolved rapidly over the past few years. In 2019, the results of phase-3 clinical trials for three disease-modifying therapies (DMT) for NMOSD were published [1–3]. In addition to the progress in relapse prevention already achieved with existing off-label agents [4], these newer immunotherapies will likely improve the overall prognosis of NMOSD even further. Several retrospective or non-controlled studies have demonstrated the value of plasma exchange (PLEX) in combination with steroids in treating acute relapses and improving relapse outcomes in NMOSD patients [5–7]. However, clinical relapses in NMOSD tend to be severe and residual disability remains high despite optimized preventive and relapse treatment [8, 9]; hence, an important unmet need is the determination of best practices related to chronic symptomatic management and restorative therapies. Residual disability and chronic symptoms have a profound effect on patient experience and quality of life (QOL) in NMOSD patients, [10–12] but they remain understudied. The aim of this review is to describe symptomatic and potentially restorative therapies in NMOSD based on available evidence and clinical experience. We also list knowledge gained in multiple sclerosis (MS), and other conditions that affect the spinal cord or optic nerves that could potentially be used to treat patients living with NMOSD. Transient symptoms that occur in the setting of a relapse and may resolve with acute immunotherapy (e.g. intractable hiccups/vomiting) are not the focus of this review. Related conditions such as myelin oligodendrocyte glycoprotein antibody disease (MOGAD) are also not covered.

## Neuropathic pain in NMOSD

Pain is prevalent in patients with NMOSD affecting more than 85% of patients [13, 14]. Painful areas are most often around the chest and waist, along the entire length of the legs, or in the back [15]. Sensory testing often reveals touch and temperature sensory loss with ongoing pain, thermal hyperalgesia, mechanical allodynia and/or paradoxical heat sensations [16]. Often the painful dermatomes correspond to the location of the spinal cord lesions found on MRI; however, the severity of the pain does not correlate with disease duration, age, AQP4-IgG status, or the number of relapses [17, 18].

Spinal cord lesions in patients with NMOSD primarily affect the cervical and thoracic segments, mainly around the central canal and adjacent grey matter in the dorsal and ventral horns of the spinal column [17]. Approximately 10–15% of all patients with NMOSD have linear medullary lesions, which are located in the floor of the fourth ventricle and expand into the mesencephalon along the periaqueductal grey matter [19]. It is suspected that these lesions may involve central nervous system (CNS) structures that are either adjacent to or contain nociceptive or anti-nociceptive pathways, subsequently leading to pain [17]. A recent clinical-radiological cross-sectional study suggested that the thoracic cord is involved in pain generation in NMOSD while the ventral posterior thalamic nucleus is involved in modulation of pain intensity [20].

Neuropathic pruritus or itch is a special type of neuropathic pain that has been reported in 27–64% of NMOSD cohorts depending on the study [21, 22]. Neuropathic pruritus can occur before, during, or occasionally following a spinal relapse [21]. It is postulated to result from irritation of a special type of neuronal subpopulation in the dorsal root ganglia that mediate pruritus [21].

In early active lesions, it has been postulated that a multidrug treatment focusing on mitigation of neuroinflammation may provide some relief [17]. These medications include minocycline, peroxisome proliferator-activated receptor agonist, cell cycle inhibitors, statins, and progesterone [17]. Another potential treatment is ketamine or memantine, which could be used to block NMDA receptors, in order to prevent the long-term potentiation at C-fiber synapses [17]. However, neuropathic pain—including pruritus—in the setting of acute myelitis often improves with commonly employed relapse treatments (corticosteroids and PLEX) [23].

For neuropathic pain associated with chronic lesions, although not specifically studied in NMOSD, multiple medications have been studied for pain relief in patients with MS and spinal cord injury (SCI). These include antiepileptic medications, as they are known to reduce the capacity of neurons to generate high-frequency action potentials. In particular, gabapentin, pregabalin, and levetiracetam have been found to reduce pain by 50–100% on average and improved self-reported Pain Scale ratings in randomized controlled trials [24–26]. Another class of medications found to help alleviate neuropathic pain are serotonin noradrenergic reuptake inhibitors (SNRIs). For example, duloxetine in particular has been found in a large randomized controlled trial of 239 MS patients to achieve statistically significant (but clinically modest) pain reduction [27]. Subcutaneous botulinum toxin injections (BTXI) to selected painful areas have also demonstrated statistically and clinically significant pain alleviation in a randomized controlled study of 40 SCI patients [28]. Intrathecal baclofen (ITB) in one uncontrolled non-blinded study of 14 patients with post-stroke or SCI-related pain demonstrated pain relief in 9 of these patients [29]. In addition to their potential benefit against neuropathic pain, both BTXI and ITB are effective against painful tonic spasms and spasticity-related pain as will be discussed later in this review. Cannabinoids have also been found in small randomized trials to have a statistically significant (but clinically modest) pain reduction effect and an even larger impact on QOL improvement [28]. Opioids have had conflicting data as to their efficacy in the management of NMOSD-associated pain [28]. This is thought to be due, in part, to the propensity for NMOSD lesions to target areas with a high density of opiate receptors, such as the periaqueductal grey, making opioid treatment less effective [13].

Non-pharmacological treatments for central neuropathic pain have been explored. Regular moderate aerobic exercise has been found to reduce pain signs in animal models through the increase of endogenous opioid content in the brainstem. Transcutaneous electrical nerve stimulation, the “Scrambler ST-5 TENS” device in particular, has been shown to be effective in the treatment of NMOSD-associated pain with a significant reduction of pain seen within 10 days [30]. This randomized sham-controlled study provided class 2 evidence that scrambler therapy can reduce neuropathic pain in NMOSD. The spinal cord stimulator (SCS) has not been studied in NMOSD but has been shown to alleviate MS pain in multiple studies [31]. Furthermore, with respect to longevity of pain relief with SCS, in a study of 19 MS patients of which 17 had an SCS implanted, 74.1% retained significant pain relief after implantation with an average follow-up of 97 months [32]. Cognitive behavioral therapy (CBT) has conflicting data with questionable efficacy on pain. However, the consensus is that its effect is based on improvement of the response to pain as opposed to treatment of the cause of pain itself [28].

## Tonic spasms and other involuntary movements

Focal spinal cord disease can cause a variety of involuntary movements including tonic spasms, paroxysmal or fixed focal dystonia, spinal myoclonus, spontaneous or triggered clonus, spinal tremor, and secondary restless leg syndrome (RLS) [33–35]. Some of these movement disorders, in particular tonic spams and paroxysmal dystonia, are thought to be more common in NMOSD than other demyelinating diseases and they can be painful [36–39]. The prevalence of any spinal movement disorder in NMOSD is estimated at 43% while the prevalence rates for tonic spasms in particular range from 22 to 40% depending on the study [33, 36, 38]. They commonly occur several weeks to months following a spinal attack but they can occasionally be the presenting symptom of a new spinal relapse [33]. Tonic spasms and other involuntary movements have a significant impact in NMOSD and are among the most bothersome residual symptoms [33, 36, 38]. Demyelination-related involuntary movements and other paroxysmal events are thought to result from ephaptic transmission of nerve impulses in the demyelinated fibers and are believed to respond to standard doses of anti-seizure medications especially sodium channel blockers like carbamazepine and oxcarbazepine [33, 34]. Tonic spasms result from inflammation and demyelination of the pyramidal tracts in the spinal cord and can occur in the absence of or before the development of tonic spasticity [34]. They may resolve on their own after recovery from a spinal attack but may re-emerge when tonic spasticity sets in [40].

In a case series of 37 NMOSD patients, 43% reported tonic spasms and other spinal involuntary movements. Of those, 88% reported partial improvement in their involuntary movements with a combination of anti-seizure medications (carbamazepine, oxcarbazepine, gabapentin, and/or pregabalin) and muscle relaxants (baclofen and/or tizanidine) 0.3 [3] Full or near full response was reported by three patients, one treated solely with carbamazepine, one treated with ITB, and one with BTXI (for cervical dystonia). The involuntary movement eventually recurred except in the patient treated with carbamazepine [33].

In observational studies focusing solely on tonic spasms, phenytoin, carbamazepine or oxcarbazepine seemed to fare better than gabapentin and muscle relaxants in controlling spasms but most patients required long-term therapy to maintain control [36, 41]. In a large retrospective NMOSD cohort from China, most patients (71–100%) with simple tonic spasm subtypes (flexor, extensor, adductor, and isometric) and 57% of those with complex tonic spams improved with a treatment regimen based on sodium channel blockers (carbamazepine, oxcarbazepine) as a first-line and anti-depressants (venlafaxine, amitriptyline) as a second-line [38].

In patients who are resistant or intolerant to carbamazepine, a few case reports have suggested benefit from topiramate [42], lacosamide [43], and ITB. MS studies and case series also support ITB as well as BTXI and levetiracetam in the treatment of refractory tonic spasms [44–46].

## Spasticity and other muscle tone abnormalities

Spasticity is an abnormal increase in muscle tone secondary to lesions affecting the descending corticospinal and parapyramidal tracts [47]. It has a constant “tonic” component and an episodic “phasic” component. The tonic component leads to limb stiffness and hypokinesia while the phasic component results in tonic spasms and several other hyperkinetic movements [40]. As mentioned previously, inflammation and demyelination of the pyramidal tracts in the spinal cord in NMOSD patients can result in tonic spasms and other involuntary movements (phasic spasticity) even before the development of the constant tonic phase [33, 34]. In this section, we will focus primarily on tonic spasticity.

There are no definitive estimates of spasticity in NMOSD but evaluation of 522 self-identified NMOSD patients from the “Patients-Like-Me” online community suggested a prevalence rate of 52% [48]. This is higher than the 37% estimated rate in some MS studies [49], which is likely reflective of the higher frequency of motor spinal attacks in NMOSD compared to the sensory-predominant attacks in MS [8]. Spasticity can impair residual function, cause pain, limit the range of motion, and predispose to skin breakdown and infections [50]. We were not able to identify any dedicated studies of tonic spasticity management in NMOSD. Based on MS studies and clinical experience, three major approaches are commonly utilized [44]. The first approach is the use of oral anti-spasticity agents including the GABA-B agonist baclofen and/or the α2 agonist tizanidine. Both were studied in randomized controlled trials and are effective against tonic spasticity in MS but can cause excessive sedation and occasionally interfere with motor function. Patients who rely on their spasticity to compensate for lower extremity weakness can experience gait worsening with these muscle relaxants. Other less commonly used oral agents for spasticity include the peripherally acting dantrolene and cannabinoid receptor agonists. The latter group has shown modest but controversial evidence in randomized controlled trials of MS-related spasticity [51] and their general use in NMOSD is not evidence based. The use of benzodiazepines (GABA-A modulators) for NMOSD-related spasticity is also controversial because of the implication of GABA-A enhancement in the overexpression of AQP4 water channels on the surface of astrocytes potentially increasing the risk of relapse [52].

An alternative approach to spasticity management involves the use of local BTXI to areas of focal spasticity [44]. Focal spasticity in NMOSD and other demyelinating disorders manifest in the form of spastic postures or focal spastic dystonia [33, 34, 40]. Muscle selection depends on the specific posture. Common spastic postures include torticollis or laterocollis, flexed elbow, clinched fist, tight Achilles tendon, and flexed toes. Toxin-based therapy is generally not suitable for diffuse spasticity because the dose needed to cover multiple large muscles would exceed safety limits. Randomized controlled trials of BTXI were successful against MS spasticity [44].

For refractory cases, the use of ITB via an implanted subcutaneous pump may be indicated [38]. ITB has not been studied in NMOSD, but based on randomized controlled MS studies, ITB reduces tonic and phasic spasticity, alleviates pain, and improves QOL [31, 44]. It is indicated in patients with diffuse spasticity who cannot tolerate oral agents or in whom oral agents are ineffective at tolerable doses. It can be used in non-ambulatory patients at high infusion rates to reduce or eliminate hypertonia to facilitate care and prevent skin breakdown. In ambulatory patients, lower infusion rates are commonly used to improve spasticity without negatively affecting the ability to walk. A pre-implantation test bolus of ITB, delivered via intrathecal injection, is usually given to determine the response and possible adverse effects in each patient. Possible but preventable side effects of an implanted ITB pump include excessive weakness, baclofen overdose, and baclofen tolerance (a constant need for higher infusion rates). However, the main risk associated with the use of ITB is the potential for baclofen withdrawal in case of missed refill appointment, catheter leak, or programming errors [53]. Hardware related complications include infection, spinal catheter migration or kinking, and skin erosions.

Combining more than one or all three anti-spasticity approaches may be needed in some NMOSD patients to relieve severe spasticity and tonic spasms. Pharmacological therapy should be coupled with daily stretching, exercise, and physical therapy.

In rare cases, NMOSD may be associated with hypotonia (or mixed hypertonia and hypotonia) secondary to extensive involvement of the anterior horn cells, severe spinal cord atrophy, and/or the rare association with autoimmune polyneuroradiculopathy [54–56]. There is currently no known treatment for hypotonia but clinicians should eliminate any unnecessary anti-spasticity agents in NMOSD patients with symptomatic hypotonia or avoid high doses in patients with mixed hyper and hypotonia. Muscle strengthening exercise and physical therapy should be attempted.

## Bladder, bowel and sexual dysfunction in NMOSD

Bladder dysfunction in NMOSD is a common symptom that affects QOL and increases morbidity. In a cross-sectional study of 14 NMOSD patients and 34 MS patients, lower urinary tract dysfunction was found to be more severe in NMOSD compared to MS [57]. Furthermore, it was determined that lower urinary tract dysfunction in patients with NMOSD might occur independently from any other neurologic disabilities [57]. In another cross-sectional analysis, in a cohort of 60 NMOSD patients, 47 patients (78%) reported bladder symptoms [58]. The most bothersome life sequela due to these urinary symptoms were, feeling worn out (89%), travel restrictions (77%), need to frequently change undergarments (74%), and limitation on performing daily activities (70%) [8] Beyond these effects on QOL, are the significant healthcare expenditures that result from recurrent urinary tract infections, sacral ulcer development, and prolonged hospital stays [59].

Based on urodynamic studies from 23 NMOSD patients during the initial phase of an attack, urinary retention was the predominant finding [59]. After resolution of this “spinal shock” episode, patients often develop symptoms of increased frequency, urgency, nocturia, and incontinence over the following 4–6 weeks [59]. In the same study, urodynamics were performed in 42 NMOSD patients in remission. The most common urodynamic findings were non-contractile detrusor (47.6%) and neurogenic detrusor overactivity with (33.3%) or without (19%) detrusor-sphincter dyssynergia [59]. Thus, patients can have components of urinary retention mixed with urinary incontinence. Furthermore, it was noted that despite complete resolution of neurologic symptoms in some patients, no NMOSD patients had normal urodynamic studies, indicating that there is no definitive correlation between sensorimotor recovery and resolution of autonomic dysfunction [59].

As bladder dysfunction in both MS and NMOSD is usually due to spinal cord lesions, it has been postulated that bladder dysfunction in NMOSD can be treated in a similar way to MS [60]. Initial non-pharmacological treatments include bladder retraining, timing of fluid intake, and pelvic floor exercise [60]. If non-pharmacological options do not provide adequate symptom control, the mainstay treatment options for incontinence are anti-muscarinic drugs (e.g. oxybutynin, darifenacin or solifenacin) or selective beta-3 adrenergic receptor agonists (e.g. mirabegron). These agents were all studied in positive randomized controlled studies in patients with mixed-cause overactive bladder including a subset of patients with MS [61–63]. These may be used in combination with desmopressin and clean intermittent self-catheterization if post-void residuals are elevated [64].

If these options fail, neuromodulation therapies have shown some success in bladder management [31]. Spinal cord stimulation has objectively been shown to increase bladder capacity and improve sphincter control in 42.5% of 40 MS patients [65]. Alternatively, posterior tibial nerve stimulation (PTNS), which modulates the L4–S3 nerve roots, has been shown to increase mean urine volume and maximal cystometric capacity, while suppressing detrusor contraction in MS patients with overactive bladder [66]. Sacral neuromodulation (SNM) involves stimulating the pelvic nerves with an implantable pulse generator, and has been shown to improve urinary symptoms in medically-refractory overactive bladder [67]. In a trial of SNM in a cohort of 17 MS patients, a long-lasting QOL improvement with a decrease in number of self-catheterizations was seen in 75% of patients [63]. SNM and PTNS are not recommended for bladder hypoactivity [68]. Detrusor BTXI are possible adjunctive therapies that could be considered in patients with neurogenic detrusor overactivity [60]. If bladder dysfunction is refractory to all available treatment options, a long-term urethral or suprapubic indwelling catheter should be offered as this may prevent recurrent pyelonephritis and kidney dysfunction [60] .If an indwelling catheter is used, it is important to institute measures to reduce the risk of infection like urine acidification and periodic catheter replacement.

Bowel dysfunction is as common as bladder dysfunction in NMOSD affecting nearly 78% of patients [58]. It usually manifests with constipation and less frequently with fecal incontinence [58] .No specific studies have been conducted for NMOSD-related bowel dysfunction but the common practice is to start with dietary fibers, laxatives, stimulants, and stool softeners. Severe or complex bowel dysfunction including fecal incontinence may respond to SNM or PTNS [31]. In refractory cases, a colostomy may be necessary [69].

Sexual dysfunction is very common in NMOSD affecting about 43% of sexually active females and 75% of sexually active males according to one study [70]. No specific treatments for sexual dysfunction in NMOSD have been formally tested but phosphodiesterase inhibitors (e.g. sildenafil) may be of benefit in treating erectile dysfunction in males and sexual arousal disorder in females if not contraindicated for cardiac or vascular reasons [71, 72].

## Fatigue and sleep disorders

Fatigue is a common symptom in NMOSD affecting nearly 70% of patients [73]. It has been associated with poor QOL, depression, and pain [73–75]. Despite study of this symptom in MS and NMOSD, the nature and origin of fatigue remains poorly understood.

Patients’ definition of fatigue varies from one to another. Physical fatigue, mental fatigue, sleepiness, and low mood are all considered forms of fatigue. In general, physical fatigue is thought to be more prevalent in NMOSD than mental fatigue, possibly due to the relative cortical sparing in NMOSD compared to MS [76]. Certain brain and/or spinal anatomical locations have been linked to physical fatigue but studies have not been consistent [73, 77, 78]. A pilot study showed improvement in fatigue scales in nine NMOSD patients treated with the IL-6 inhibitor tocilizumab as a preventive therapy [79]. However, this positive impact on fatigue was not replicated in the phase-3 double-blind trial of the longer acting IL-6 inhibitor satralizumab [3]. In one small study, treatment with levocarnitine did not improve fatigue in a subgroup of NMOSD patients with low levocarnitine levels [80]. Apart from these three studies, there have not been any other published studies evaluating treatment of fatigue in NMOSD to our knowledge. In clinical practice, treatment of fatigue is often based on insights from MS literature, which has not been conclusive in this area. A thorough evaluation of correctable causes of fatigue should take place in every case. Adequate treatment of sleep disorders and/or depression can improve fatigue. Elimination of unnecessary sedating medications or reduction of their dose can be helpful in some patients. Non-pharmacological approaches to fatigue treatment (e.g. light exercise, aquatic therapy) should be utilized before pharmacological treatment as they have been shown to be effective in MS fatigue [81, 82], Pharmacological options include amantadine, modafinil, and methylphenidate [83–85]. These agents have shown mixed results in MS studies but in clinical practice, some patients appear to be responsive to one or more of these agents. However, a recent double-blind randomized placebo-controlled study in MS patients showed that neither of these three agents had benefit over placebo in ameliorating fatigue [86]. Online-based self-administered cognitive-behavioral interventions against fatigue have shown benefit in MS patients [87], and so did transcranial magnetic stimulation in a number of small studies [31, 88].

A variety of sleep disorders can be seen with NMOSD. Acquired narcolepsy and hypersomnia can present acutely secondary to diencephalic relapses and often resolve with acute anti-inflammatory therapy or by virtue of time [89, 90]. However, permanent hypothalamic damage is possible and can lead to decreased orexin levels with subsequent excessive daytime sleepiness and chronic narcolepsy [91, 92]. Symptomatic treatment with modafinil can be tried in symptomatic patients if needed. In addition to sleep disorders secondary to diencephalic dysfunction, NMOSD patients can also experience higher rates of obstructive sleep apnea (OSA), RLS, impaired sleep structure, and poor sleep quality than healthy control and MS patients [93, 94]. The use of continuous positive airway pressure during sleep should be encouraged in NMOSD patients with OSA to improve sleep quality and fatigue. Secondary RLS usually follows spinal attacks in NMOSD and it can be responsive to dopamine agonists or gabapentin [34, 95].

## Psychiatric and cognitive impairment

Several studies have shown that depression, suicidality, and cognitive impairment are more prevalent in NMOSD patients compared to healthy controls [96–98]. It is unclear if these symptoms are related to direct brain involvement or secondary to the psychological burden of the neurologic disability in NMOSD patients. Although no specific studies have addressed management of depression in NMOSD, evidence from MS studies suggest a role for CBT, with or without selective serotonin reuptake inhibitors, for management of mood disorders [99]. Evidence from MS studies also suggests that cognitive rehabilitation, aerobic exercise, and amphetamines may be helpful for cognitive impairment [100]. Streamlining medications and reduction of unnecessary polypharmacy are important and so is treatment of mood and sleep disorders. Periodic screening of cognitive function with the Symbol Digit Modalities Test or other screening tools is important for detecting new symptoms and monitoring progression of existing symptoms [100]. A meta-analysis of randomized controlled trials showed no convincing benefit from cholinesterase inhibitors and memantine for MS-related memory disorder [101]. More research is needed to better characterize mood and cognitive disorders in NMOSD to elucidate the pathogenesis and best therapeutic interventions in this patient population.

## Motor and visual dysfunction

Overall, 34% of NMOSD patients develop permanent motor dysfunction, with 23% of these patients becoming wheelchair-bound after a median disease duration of 75 months [102]. The mainstay of treatment of motor weakness is neurorehabilitation. The goal of rehabilitation in NMOSD is to prevent complications, treat symptoms and recover functionality to minimize the handicap remaining after relapse. There is limited data on the utility of rehabilitation in patients with NMOSD but it is current expert consensus that comprehensive therapy is helpful in returning elements of functionality to patients [103]. However, the extent of functional recovery may be limited in comparison to other forms of SCI mainly because NMOSD lesions are longitudinally extensive, affecting multiple spinal levels and because there is often damage to the anterior horn cells [104].

In a retrospective study comparing the effect of inpatient rehabilitation on patients with NMOSD and MS, intensive therapy five days a week, including physiotherapy, occupational therapy, hydrotherapy, and psychology assessments, conferred increased functional improvement in the NMOSD cohort compared to MS [105]. The reason for why these patients showed greater improvement in functionality was attributed to the relative cortical sparing in NMOSD compared to MS allowing more active participation in rehabilitation. Functional electrical stimulation-based therapy has been shown to stabilize disability in patients with MS and to promote proliferation of progenitor cells in animal models [106, 107]. Likewise, oscillating field stimulation has been postulated as a means to induce differentiation of oligodendrocyte precursor cells (OPCs) after SCI [108]. In animal models, this technique has been shown to significantly improve remyelination after SCI while also promoting locomotor recovery.

Another consideration for neurorehabilitation is the brain–computer interface (BCI), which uses immersive virtual reality with visual-tactile feedback and electroencephalogram-controlled robotic actuators as a means to train the body [109]. In a small study of eight patients with SCI, after 12 months of BCI-based training, all patients showed neurologic improvements in respect to somatic sensation and voluntary motor control [109]. Sensory BCI can also be used to restore vision in blind individuals via direct stimulation of the visual cortex by implanted electrodes [110]. This technology is still in need for refinement before being usable clinically and has not been studied in NMOSD. The relative cortical sparing in NMOSD compared to MS makes the future use of cortically based BCI techniques more plausible in NMOSD.

Dalfampridine, a potassium channel blocker approved for improving speed of walking in MS patients, was tested in a randomized controlled trial against placebo in 13 patients with transverse myelitis [111]. Although the study did not reach its primary outcome, there were trends towards improvement in the dalfampiridine arm in the 25-foot-walk test and several secondary outcomes [111]. A non-controlled study of dalfampiridine in 14 NMOSD patients was presented in 2015 (not published in a peer-reviewed journal at the time of writing) demonstrating positive impact on walking speed, fatigue, and QOL [112]. Further studies are needed to confirm these preliminary findings in the NMOSD population.

Most importantly, remyelination and neuroregeneration are the principle therapeutic goals for motor and visual dysfunction in NMOSD. Like many other neurological disorders, regenerative research in NMOSD is limited and lags behind immunomodulation research. The recent success in immune-modulating therapies in NMOSD might stir interest in regenerative research in the coming years. In the next section, we will provide an overview of the preliminary studies of remyelination and mesenchymal stem cell (MSC) therapy in NMOSD.

## Reparative and remyelination therapies

The AQP4 water channel, the main pathologic target of AQP4-IgG, is located on the surface of astrocytes. Therefore, NMOSD is viewed as a primary astrocytopathy with secondary bystander injury to the oligodendrocytes and subsequent demyelination and axonal loss [113]. Besides the secondary widespread demyelination, remyelination mechanisms also seem to be dysfunctional in individuals with NMOSD. CSF levels of glial fibrillary acidic protein (GFAP), an intermediate filament protein of the astrocyte cytoplasm, is considered a good surrogate to measure astrocyte damage and clinical severity in NMOSD [114]. Myelin basic protein (MBP) is a biomarker for active demyelination and neurofilament light chain (NFL) indicates axonal damage in neuroinflammatory diseases. GFAP levels are found to be higher in NMOSD when compared to MS, whereas MBP and NFL levels were not significantly distinctive from MS [108]. However, while GFAP levels rapidly return to normal following treatment for an NMOSD attack, MBP remains high, suggesting that despite the acutely predominant astrocytic damage in NMOSD, active demyelination persists for longer [115]. Furthermore, AQP4-IgG-positive individuals demonstrate the downregulation of CXC-motif ligand 12(CXCL12), a chemokine protein released by astrocytes that plays a role in the activation of OPCs to promote remyelinating processes [116]. A more recent study found that NFL levels are higher in patients with NMOSD in comparison with MS and MOGAD suggesting prominent axonal injury in NMOSD*.*[117]

The outlook of reparative therapies in NMOSD will likely follow novel treatment approaches in MS, as the prototypical CNS demyelinating disease. Currently, several strategies to counteract the neurodegenerative consequences of chronic demyelination in MS are being investigated [118].

In essence, potential agents seek to amplify the endogenous remyelinating mechanisms of the CNS mainly by targeting the recruitment and stimulation of OPCs. A study aimed at evaluating the efficacy of 14 potential remyelinating drugs showed that only clobetasol, an FDA-approved topical corticosteroid, demonstrated capability for promoting OPC maturation and remyelination both in ex vivo cerebellar culture model and in vivo mouse model of NMOSD [119]. Intraperitoneal administration of clobetasol in mice significantly reduced myelin loss by approximately 60%, an effect that seems to be derived by its ability to increase the number of mature differentiated oligodendrocytes around NMOSD lesions. It is thought that activity of clobetasol on OPCs is mediated by glucocorticoid receptor signaling modulation, which is known to be important in myelin gene expression in the CNS. Its effectiveness in promoting functional remyelination in vivo had been previously proven in MS [120]. These results were considered as proof-of-concept for the potential utility of remyelinating therapies in NMOSD.

Multiple additional remyelinating therapies for MS are currently under different stages of development. Besides clobetasol, laboratory screening methods have identified several other molecules that could potentially enhance the function of endogenous OPCs including miconazole, clemastine fumarate, N-acetyl glucosamine, and benztropine [121]. Of these, clemastine (a first generation antihistamine) has been evaluated in a single-center, randomized, controlled phase-II trial, with apparent improvement in visual evoked potential measurements in MS [122]. Other remyelination-promoting strategies under clinical trial evaluation include high dose biotin, anti-LINGO-1 antibodies (i.e. opicinumab), and MSC transplantation [118]. In a double-blind randomized placebo-controlled pilot trial, 12.6% of patients with progressive MS receiving high dose biotin had disability improvement compared to none in the placebo arm (*P* < 0.05) [123]. However, a similar study looking at reversal of visual disability in MS patients with high dose biotin did not achieve its primary outcome [124]. Recently, a phase-3 double-blind placebo-controlled study of high dose biotin in progressive MS failed to achieve primary outcome of disability improvement [125]. Preliminary studies of opicinumab have shown modest potential for improvement in neurological function in MS patients [126]. However, when considering the repurposing of remyelination strategies from other demyelinating diseases, it is important to consider the evidence that repair mechanisms of MS and NMOSD may be significantly different. Astrocyte recovery, OPCs stimulation and oligodendrocyte repopulation in NMOSD might not be sufficient for effective remyelination of axons [127]. Since NMOSD pathogenic mechanisms are particularly diverse and involve a composite of astrocyte, oligodendrocyte, microglial, neuronal and peripheral immune cell dysfunction, successful remyelination might also be derived from a combination of several processes, rendering it particularly difficult to tackle. Despite our rapidly growing knowledge, there are still many questions on the exact mechanisms behind the apparently impaired oligodendrocyte maturation, as well as the behavior of the newly regenerated astrocytes and persistent microglial dysfunction. Understanding these disease-specific obstacles to remyelination is crucial in the development of new treatments [128].

## Mesenchymal stem cell therapy

Mesenchymal stem cells (MSC) are pluripotent, non-hematopoietic cells residing in many tissues, but are commonly isolated from the bone marrow and adipose tissue. MSC are able to differentiate into several cell lineages, secrete a wide variety of growth factors, and have been demonstrated to have immunomodulatory, reparative, and tissue-protective properties [129]. For therapeutic use, following collection, MSC are standardly culture-expanded to increase cell number to required dosage and to ensure cell type purity. Therapeutic delivery of MSC is often through intravenous or intrathecal routes for systemic or CNS-specific administration, and through matrix embedding or injection for local needs.

MSC are thought to act beyond the immediate area through the paracrine effects of the secretome factors that they produce [130]. In addition to their reparative potential, the immunomodulatory effects of MSC may impact NMOSD by modulating both the innate and adaptive immune system through suppression of many functions of T, B, and natural killer cells, which have been shown to be critical in NMOSD pathogenesis [131].

For NMOSD, MSC are not commercially available and are used on experimental basis only. There have been a few small studies investigating the safety and efficacy of MSC in NMOSD reported to date. A trial of intravenous autologous MSC infusion in 15 NMOSD patients showed sustained improvement in neurological disability over a 2 year period without major adverse events [132]. Therapeutic improvement of five seropositive NMOSD patients treated with human umbilical cord-derived MSC (hUC-MSC) has been reported [133]. Through the duration of the 24 month study, four of the five patients demonstrated improved disability scores, and all showed a significant improvement in relapse numbers. A 10 year follow-up showed no additional adverse events [134].

The regenerative potential of MSC was evident in a report of autologous bone-marrow-derived, culture-expanded MSC (BM-MSC) administered locally in a collagen matrix to treat extensive pressure ulcers on the legs of an NMOSD patient with paraplegia [135]. Healing of the pressure ulcers occurred within 8 days, followed by progressive disability improvement. Notably, gait function improved such that the patient was able to walk with bilateral assistance within 2 months and without assistance within 1 year. The patient remained in a sustained remission and relapse free state for 6 years. Additional studies should be performed to assess the safety and efficacy of BM-MSC in NMOSD. A recent phase-2 study demonstrated benefit of intravenous and intrathecal MSC in patients with progressive MS including favorable effect on disease progression and walking speed [136].

Autologous MSC are often preferred over allogeneic as they decrease the possibility of infection spread and potential tissue rejection issues. The dose needed for therapeutic efficacy is often well above the amount easily extracted from the bone marrow or adipose tissue from a donor, so they are usually culture-expanded prior to administration. This is not problematic if the MSC of an individual with a specific disease are not affected by the disease state. BM-MSC from donors with NMOSD proliferated at a lower rate compared to healthy controls and also had a higher rate of cell death in one study [137]. This observation is concerning for the potential use of autologous MSC in treating NMOSD. The authors posited that the addition of the proliferation agonist platelet-derived growth factor may counteract the low proliferation rate such that the use of autologous MSC from NMOSD patients may become feasible, but these data need to be considered for any future studies. The best delivery and processing methods of MSC are yet to be elucidated and larger studies are needed to explore the full potential of this intervention in NMOSD.

## Summary

There is a paucity of data on symptomatic and restorative therapies in NMOSD. Most interventions are based on small studies in the NMOSD population or rely on evidence from MS or other neurological disorders. The relative rarity of NMOSD hinders large-scale clinical trials but the recent success of multi-center collaborative DMT trials provides a basis for similar future efforts focusing on symptomatic, restorative and regenerative medicine. The relative higher severity of residual symptoms and neurological disability in NMOSD compared to MS makes the need for timely symptomatic and regenerative trials in NMOSD more urgent. Until better evidence becomes available, we hope that the information summarized in this review will provide some guidance to clinicians caring for NMOSD patients and provide direction for future research. Table 1 lists some of the most commonly used pharmacological symptomatic therapies in NMOSD. Table 2 contains suggested areas for future research pertaining to each of the topics covered in this review.

**Table 1:** Pharmacological options for symptomatic therapy in NMOSD

Symptomatic therapy	Indications	Common daily dosage	Side effects	Class of evidence*
Anti-convulsants				
Gabapentin	-Neuropathic pain -Tonic spasms	300–3600 mg	Dizziness, drowsiness, fatigue, falls	Class 1 for SCI-related neuropathic pain [1]
Pregabalin	-Neuropathic pain -Tonic spasms	50–300 mg	Dizziness, drowsiness, fatigue, falls	Class 1 for SCI-related neuropathic pain [2]
Carbamazepine	-Tonic spasms -Neuropathic pain	100–1200 mg	Dizziness, drowsiness, nausea, vomiting, ataxia, hyponatremia, agranulocytosis, skin rash	Class 4 for NMOSD-related tonic spasms [3]
Oxcarbazepine	-Tonic spasms -Neuropathic pain	150–1200 mg	Dizziness, drowsiness, nausea, vomiting, ataxia, hyponatremia, agranulocytosis, skin rash	Class 4 for NMOSD-related tonic spasms Class 1 for SCI-related neuropathic pain [4]
Levetiracetam	-Neuropathic pain	250–1000 mg	Irritability, agitation, drowsiness	Class 1 for MS-related neuropathic pain [5]
Muscle relaxants				
Oral baclofen	-Spasticity -Tonic spasms	5–80 mg	Sedation, dizziness, drowsiness, nausea, vomiting, urine retention	Class 2 for MS-related spasticity Class 3 for MS-related tonic spasms [6]
Intrathecal baclofen	-Spasticity -Tonic spasms	100–2000 mcg	Muscle weakness, risk of baclofen withdrawal syndrome, sedation	Class 1 for MS-related spasticity and tonic spasms [6]
Tizanidine	-Spasticity -Tonic spasms	2–36 mg	Sedation, dizziness, drowsiness, nausea, livery injury	Class 1 for MS-related spasticity [6]
Dantrolene	-Spasticity	25–400 mg	Weakness, hepatotoxicity	Class 3 for MS-related spasticity [6]
Botulinum toxin injections	-Focal spasticity or dystonia -Overactive bladder -Tonic spasms -Neuropathic pain	50–300 units	Depend on the site of injection and may include: focal weakness, dysphagia, dry mouth, urinary retention, rarely generalized weakness	Class 1 for MS-related spasticity [6] Class 1 for SCI-related neuropathic pain [7]
Anti-depressants				
Duloxetine	-Neuropathic pain -Depression and anxiety	30–120 mg	Nausea, somnolence, hypertension, liver injury, serotonin syndrome	Class 1 for MS-related neuropathic pain [8]
Venlafaxine	-Neuropathic pain -Depression and anxiety	37.5–225 mg	Nausea, somnolence, hypertension, liver injury, serotonin syndrome	Class 2 for SCI-related neuropathic pain [9]
Amitriptyline	-Neuropathic pain -Depression and anxiety	12.5–150 mg	Sedation, dry mouth, constipation	Class 2 for SCI-related neuropathic pain [10]
Stimulants				
Modafinil	-Narcolepsy -Fatigue	100–200 mg	Headache, insomnia, hallucinations, addiction	Class 1 for narcolepsy [11]
Amantadine	-Fatigue	100–300 mg	Nausea, dizziness, insomnia, constipation, dry mouth, hallucinations	Class 4 for MS-related fatigue (class 1 that it’s no better than placebo for MS-fatigue) [12]
Medications for bladder dysfunction				
Oxybutynin	-Overactive bladder	5–30 mg	Dry mouth, constipation, urine retention, cognitive decline	Class 1 for MS-related overactive bladder [13]
Darifenacin	-Overactive bladder	7.5–15 mg	Dry mouth, constipation, urine retention, cognitive decline	Class 1 for overactive bladder [14]
Solifenacin	-Overactive bladder	5–10 mg	Dry mouth, constipation, urine retention, cognitive decline	Class 1 for MS-related overactive bladder [13]
Mirabegron	-Overactive bladder	25–50 mg	Hypertension, constipation, urine retention, urinary tract infections	Class 1 for MS-related overactive bladder [15]
Medications for walking impairment				
Dalfampridine	-Walking impairment	20 mg	Insomnia, dizziness, headache, urinary tract infections, seizures	Class 1 for MS-related walking impairment [16] Class 2 for NMOSD-related walking impairment[17]

^*^See appendix for a list of references supporting class of evidence for each agent

**Table 2:** Suggested topics for future research in NMOSD symptomatic and restorative therapy

Research area	Suggested topics
Neuropathic pain	1-Head to head randomized controlled studies of anti-neuropathic pain agents in NMOSD-related pain 2-Replication of the transcutaneous electrical nerve stimulation study 3-Study of spinal cord stimulation for NMOSD-related pain
Spasticity and tonic spasms	1-Head to head randomized controlled studies of muscle relaxants and anti-seizure agents in NMOSD-related spasticity and tonic spasms 2-Study of cannabinoids in NMOSD-related spasticity and tonic spasms 3-Study of botulinum toxin for NMOSD-related tonic spasms and focal spasticity/dystonia 4-Study of intrathecal baclofen delivery for NMOSD-related spasticity and tonic spasms
Bladder, bowel, and sexual dysfunction	1-Head to head randomized controlled studies of anti-muscarinic drugs and selective beta-3 adrenergic receptor agonists in NMOSD-related bladder dysfunction 2-Study and comparison of sacral neuromodulation and posterior tibial nerve stimulation in NMOSD-related bladder and bowel dysfunction 3-Study of phosphodiesterase inhibitors and other sexual therapies in NMOSD-related sexual dysfunction
Fatigue and sleep disorders	1-Head to head randomized controlled studies of amantadine, bupropion, modafinil, and amphetamines in NMOSD-related fatigue and sleep disorders 2-Study of non-pharmacologic approaches to fatigue including aerobic exercise, and water therapy
Psychiatric and cognitive impairment	1-Longitudinal neuropsychological studies of cognitive and psychiatric dysfunction in NMOSD with radiological correlation 2-Study of cognitive rehabilitation in NMOSD-related cognitive dysfunction 3-Head to head randomized controlled studies of anti-depressants in NMOSD-related depression and/or anxiety
Motor dysfunction and walking impairment	1-Study of functional electrical stimulation-based rehabilitation in NMOSD-related motor dysfunction 2-Study of immersive virtual reality in NMOSD rehabilitation 3-Placebo-controlled randomized study of dalfampridine in NMOSD-related walking impairment 4-Study of remyelinating agents in NMOSD-related motor dysfunction 5-Study of mesenchymal stem cells in NMOSD related motor dysfunction
Visual dysfunction	1-Study of visual rehabilitation and occupational therapy for NMOSD-related visual impairment 2-Study of sensory brain-computer interface for visual cortex stimulation 3-Study of remyelinating agents in NMOSD-related visual impairment 4-Study of mesenchymal stem cells in NMOSD related visual impairment
Remyelination	1-Placebo-controlled randomized trials of clobetasol, miconazole, benztropine, clemastine, high dose biotin, and opicinumab in NMOSD-related motor and/or visual dysfunction
Mesenchymal stem cells (MSC)	1-Study of intravenous versus intrathecal MSC therapy for NMOSD-related motor and/or visual dysfunction 2-Study of human umbilical versus autologous bone marrow-derived MSC in NMOSD-related motor and/or visual dysfunction 3-Study of platelet-derived growth factor in autologous MSC therapy in NMOSD-related motor and/or visual dysfunction

## Supplementary Information

Below is the link to the electronic supplementary material.

Supplementary file1 (DOCX 16 KB)
