# Supplementary material for: Symptomatic and restorative therapies in neuromyelitis optica spectrum disorders
Source: J Neurol. 2021 Sep 5;269(4):1786–801. doi: 10.1007/s00415-021-10783-4 (PMC8940781; doi:10.1007/s00415-021-10783-4)
Supplement: Supplementary file 1 — Supplementary file1 (DOCX 16 KB) [file 415_2021_10783_MOESM1_ESM.docx]

**Appendix: List of references for table 1:**

1. Levendoglu, Funda, et al. "Gabapentin is a first line drug for the treatment of neuropathic pain in spinal cord injury." Spine29.7 (2004): 743-751.
2. Siddall PJ, Cousins MJ, Otte A, Griesing T, Chambers R, Murphy TK. Pregabalin in central neuropathic pain associated with spinal cord injury: a placebo-controlled trial. Neurology. 2006 Nov 28;67(10):1792-800. doi: 10.1212/01.wnl.0000244422.45278.ff. PMID: 17130411.
3. Li QY, Wang B, Yang J, Zhou L, Bao JZ, Wang L, Zhang AJ, Liu C, Quan C, Li F. Painful tonic spasm in Chinese patients with neuromyelitis optica spectrum disorder: Prevalence, subtype, and features. Mult Scler Relat Disord. 2020 Jul 17;45:102408. doi: 10.1016/j.msard.2020.102408. Epub ahead of print. PMID: 32712462.
4. Min K, Oh Y, Lee SH, Ryu JS. Symptom-Based Treatment of Neuropathic Pain in Spinal Cord-Injured Patients: A Randomized Crossover Clinical Trial. Am J Phys Med Rehabil. 2016 May;95(5):330-8. doi: 10.1097/PHM.0000000000000382. PMID: 26368836.
5. Rossi S, Mataluni G, Codecà C, Fiore S, Buttari F, Musella A, Castelli M, Bernardi G, Centonze D. Effects of levetiracetam on chronic pain in multiple sclerosis: results of a pilot, randomized, placebo-controlled study. Eur J Neurol. 2009 Mar;16(3):360-6. doi: 10.1111/j.1468-1331.2008.02496.x. PMID: 19364364.
6. Otero-Romero S, Sastre-Garriga J, Comi G, Hartung HP, Soelberg Sørensen P, Thompson AJ, Vermersch P, Gold R, Montalban X. Pharmacological management of spasticity in multiple sclerosis: Systematic review and consensus paper. Mult Scler. 2016 Oct;22(11):1386-1396. doi: 10.1177/1352458516643600. Epub 2016 May 19. PMID: 27207462.
7. Han ZA, Song DH, Oh HM, & Chung ME (2016). Botulinum toxin type A for neuropathic pain in patients with spinal cord injury. Annals of Neurology, 79(4), 569–578. doi:10.1002/ana.24605
8. Vollmer, Timothy L., et al. "A Randomized, Double‐Blind, Placebo‐Controlled Trial of Duloxetine for the Treatment of Pain in Patients with Multiple Sclerosis." Pain Practice 14.8 (2014): 732-744.
9. Richards JS, Bombardier CH, Wilson CS, Chiodo AE, Brooks L, Tate DG, Temkin NR, Barber JK, Heinemann AW, McCullumsmith C, Fann JR. Efficacy of venlafaxine XR for the treatment of pain in patients with spinal cord injury and major depression: a randomized, controlled trial. Arch Phys Med Rehabil. 2015 Apr;96(4):680-9. doi: 10.1016/j.apmr.2014.11.024. Epub 2014 Dec 17. PMID: 25527253.
10. Agarwal N, Joshi M. Effectiveness of amitriptyline and lamotrigine in traumatic spinal cord injury-induced neuropathic pain: a randomized longitudinal comparative study. Spinal Cord. 2017 Feb;55(2):126-130. doi: 10.1038/sc.2016.123. Epub 2016 Aug 16. PMID: 27527240.
11. Broughton RJ, Fleming JA, George CF, Hill JD, Kryger MH, Moldofsky H, Montplaisir JY, Morehouse RL, Moscovitch A, Murphy WF. Randomized, double-blind, placebo-controlled crossover trial of modafinil in the treatment of excessive daytime sleepiness in narcolepsy. Neurology. 1997 Aug;49(2):444-51. doi: 10.1212/wnl.49.2.444. PMID: 9270575.
12. Nourbakhsh B, Revirajan N, Morris B, Cordano C, Creasman J, Manguinao M, Krysko K, Rutatangwa A, Auvray C, Aljarallah S, Jin C, Mowry E, McCulloch C, Waubant E. Safety and efficacy of amantadine, modafinil, and methylphenidate for fatigue in multiple sclerosis: a randomised, placebo-controlled, crossover, double-blind trial. Lancet Neurol. 2021 Jan;20(1):38-48. doi: 10.1016/S1474-4422(20)30354-9. Epub 2020 Nov 23. PMID: 33242419.
13. Amarenco G, Sutory M, Zachoval R, Agarwal M, Del Popolo G, Tretter R, Compion G, De Ridder D. Solifenacin is effective and well tolerated in patients with neurogenic detrusor overactivity: Results from the double-blind, randomized, active- and placebo-controlled SONIC urodynamic study. Neurourol Urodyn. 2017 Feb;36(2):414-421. doi: 10.1002/nau.22945. Epub 2015 Dec 29. PMID: 26714009.
14. Zinner N, Tuttle J, Marks L. Efficacy and tolerability of darifenacin, a muscarinic M3 selective receptor antagonist (M3 SRA), compared with oxybutynin in the treatment of patients with overactive bladder. World J Urol. 2005 Sep;23(4):248-52. doi: 10.1007/s00345-005-0507-3. Epub 2005 Nov 8. PMID: 16096831.
15. Krhut J, Borovička V, Bílková K, Sýkora R, Míka D, Mokriš J, Zachoval R. Efficacy and safety of mirabegron for the treatment of neurogenic detrusor overactivity-Prospective, randomized, double-blind, placebo-controlled study. Neurourol Urodyn. 2018 Sep;37(7):2226-2233. doi: 10.1002/nau.23566. Epub 2018 Mar 31. PMID: 29603781.
16. Goodman AD, Brown TR, Edwards KR, Krupp LB, Schapiro RT, Cohen R, Marinucci LN, Blight AR; MSF204 Investigators. A phase 3 trial of extended release oral dalfampridine in multiple sclerosis. Ann Neurol. 2010 Oct;68(4):494-502. doi: 10.1002/ana.22240. PMID: 20976768.
17. Christine C, Bourre B, Mathieu B, et al., Dalfampridine for symptomatic treatment of neuromyelitis optica (P5.263). Neurology Apr 2015, 84 (14 Supplement) P5.263;
